# The Difference Quantity of Urinary Peptides between Two Groups of Type 2 Diabetic Patients with or without Coronary Artery Disease

**DOI:** 10.1155/2015/758402

**Published:** 2015-05-18

**Authors:** Guangzhen Fu, Mei Hu, Lina Chu, Man Zhang

**Affiliations:** ^1^Department of Clinical Laboratory, Peking University Ninth School of Clinical Medicine, Beijing Shijitan Hospital, Beijing 100038, China; ^2^Department of Clinical Laboratory, Capital Medical University, Beijing Shijitan Hospital, Beijing 100038, China

## Abstract

*Objectives*. We aim to explore urinary biomarkers that could monitor CAD in type 2 diabetic patients. *Materials and Methods*. Urine samples from two groups, twenty-eight type 2 diabetic patients with coexisting CAD and thirty type 2 diabetic patients without CAD, were purified by MB-WCX and then analyzed by MALDI-TOF-MS. Subsequently, we compared the urinary peptide signatures of the two groups by use of ClinProTools2.1 and evaluated the potential ability of the differently expressed peptides to distinguish type 2 diabetic patients with coexisting CAD from type 2 diabetic patients without CAD by ROC analysis. Finally, the differently expressed peptides were identified by nanoliquid chromatography-tandem mass spectrometry. *Results*. There were six differently expressed peptides (*m/z* 1305.2, 1743.9, 2184.9, 2756.1, 3223.2, and 6196.1) between the two groups of subjects, and they were identified as fragments of isoform 1 of fibrinogen alpha chain precursor, prothrombin precursor, and interalpha-trypsin inhibitor heavy chain H4. The diagnostic efficacy of *m/z* 2756.1 and *m/z* 3223.2 was better than the other peptides. Area under ROC of the *m/z* 2756.1, and *m/z* 3223.2 was 0.98 and 0.93, respectively. *Conclusions*. These urinary peptides are potential urinary biomarkers for monitoring of type 2 diabetic patients with CAD.

## 1. Introduction

Coronary artery disease (CAD) is a frequently coexisting disorder for type 2 diabetic patients and presents as a major component of public health concerns and subsequent economic burdens worldwide. Diabetes mellitus is an established risk factor for coronary heart disease [1] and confers about a twofold excess risk for a wide range of vascular diseases, independently of other conventional risk factors [2]. Early diagnosis of CAD coexisting with type 2 diabetes is a key factor for successful treatment outcome, since early detection could offer the opportunity to initiate pharmacological treatments and even measures such as prophylactic stenting, to reduce the risk of developing cardiac ischemia and myocardial infarction [3, 4]. However, the diagnosis of CAD with or without diabetes in our current clinical practices must use a variety of invasive methods (angiography for CAD) or methods that deliver radiation (coronary perfusion testing, coronary angiography, and coronary artery calcification). Therefore, improved noninvasive methods to monitor diabetes and the coexisting CAD are needed.

To this end, we focus on the urinary proteomics for they contain extensive information [5–7], and the low molecular mass proteome in urine is quite stable [8, 9]. Furthermore, it is an excellent option for the discovery of biomarkers, which can be used for the early detection, diagnosis, and therapeutic evaluation of diseases in a clinical setting.

In this study, CLINPROT MALDI-TOF MS was used to analyze the urinary peptidome profiles that could offer insight into the potential biomarkers between type 2 diabetic patients with and without CAD. Then, nanoliquid chromatography-tandem mass spectrometry was used to identify the sequence of the differently expressed peptides.

## 2. Materials and Methods

### 2.1. Study Population

This study was approved by the ethics committee of Beijing Shijitan Hospital, and the participants all gave informed consent, in accordance with the provisions of the Helsinki Declaration. The study analyzes two groups. Twenty-eight type 2 diabetic patients with coexisting clinically confirmed CAD (G, *n* = 28) and thirty type 2 diabetic patients without CAD (D, *n* = 30) from Beijing Shijitan Hospital were enrolled in this study from October 2012 to May 2013. All the type 2 diabetic patients had a fasting plasma glucose (FPG, fast for at least eight hours) ≥7.0 mmol/L and glycated hemoglobin (HbA1C) ≥6.0% (42 mmol/mol). Details of the clinical characteristics of selected subjects are shown in Table 1.

### 2.2. Urine Samples Collection and Preparation

Firstly, second void morning urine samples were collected from all the volunteers, discarding the first jet but not the final, and all volunteers were informed to refrain from unusual and heavy physical activity the day before urine collection. Moreover, the urine samples of all the selected subjects had no hematuresis, ketosis, and urinary albumin/creatinine ratio (A/Cr) less than 30 mg/g. Then, sterile polypropylene tubes were used to collect random urine samples. Immediately after collection, urine samples were centrifuged at 400 ×g for 5 minutes to remove cell debris and casts. Then we divided the supernatants into aliquots and froze them at −80°C.

The methods including fractionation of urinary peptides using weak cationic-exchange magnetic beads (Bruker Daltonics), MALDI-TOF MS AnchorChip spotting, and data acquisition were all performed as previously developed by Chu et al. [10].

### 2.3. Statistical Analyses

Descriptive patient characteristics are displayed as mean ± SD unless otherwise indicated and calculations were performed using SPSS 17.0. The peak area was used as quantitative standardization. The comparison of the peak area between two groups was performed by *t*-tests (normal distributed data) or Wilcoxon test (abnormal distributed data) using ClinProTools2.1 bioinformatics software. Two-tailed *P* values <0.05 were considered significant in all statistical comparisons. Receiver operating characteristic curve (ROC) analysis and area under the curve (AUC) calculations were constructed for determination of the diagnostic efficacy of each selected marker.

### 2.4. Peptide Sequence

A nanoliquid chromatography-tandem mass spectrometry, which consisted of an Aquity UPLC system (Waters) and a LTQ Obitrap XL mass spectrometer (Thermo Fisher) equipped with a nano-ESI source, was used to identify the sequences of differential expression peptides. Firstly the peptide solutions were loaded into a C18 trap column (symmetry 180 *μ*m × 20 mm × 5 *μ*m, nanoAcquity) with the flow rate of 15 *μ*L/min for 3 minutes. Then the desalted peptides were analyzed by C18 analytical column (symmetry 75 *μ*m × 150 mm × 3.5 *μ*m, nanoAcquity) at a flow rate of 400 nl/min. The mobile phases A (5% acetonitrile, 0.1% formic acid, Sigma-Aldrich) and B (95% acetonitrile, 0.1% formic acid) were used for analytical columns. Gradient elution profile was as follows: 5%B-45%B-80%B-80%B-5%B-5%B in 60 minutes. The MS instrument was operated in a data-dependent model. The range of full scan was 400–2000* m/z* with a mass resolution of 100,000 (*m/z* 400). The ten most intense monoisotope ions were the precursors for collision induced to two consecutive scans per precursor ion followed by 90s of dynamic exclusion.

### 2.5. Bioinformatics and Identification of Urine Biomarkers

The obtained spectra were analyzed with BioworksBrowser3.3.1 SP1 (Thermo Fisher) and the resulting mass lists were matched against the IPI Human database (v3.45) using Sequest search. Parameters were set as follows: Delton ≥ 0.1; Charge2+, Xcorr2.0; charge3+, Xcorr2.5; peptide probability ≤1*e* − 003; parent ion masses tolerance: 50 ppm; fragment ion masses tolerance: 1 Da; enzyme: no enzyme; variable modification: oxidation of methionine.

## 3. Results

### 3.1. Urinary Peptidome Profiling

**Figure 1:**
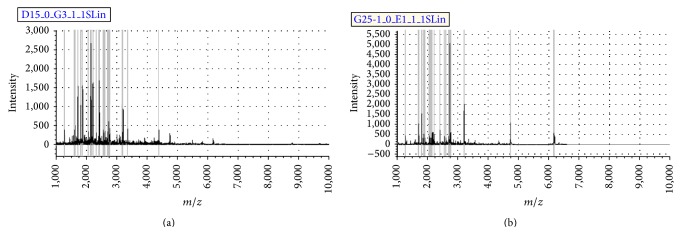
Typical urinary sample mass spectrum from MALDI-TOF MS after being purified by weak cation exchange magnetic beads. (a) From one sample of type 2 diabetic patients without coronary artery disease. (b) From one sample of type 2 diabetic patients with coronary artery disease.

Urine samples from fifty-eight volunteers purified by magnetic beads exhibited spectral peaks in the 1000–10,000 Da range. After analysis of MALDI-TOF MS, typical WCX spectra were shown in Figure 1.

### 3.2. Statistical Data Analysis between the Two Groups

**Figure 2:**
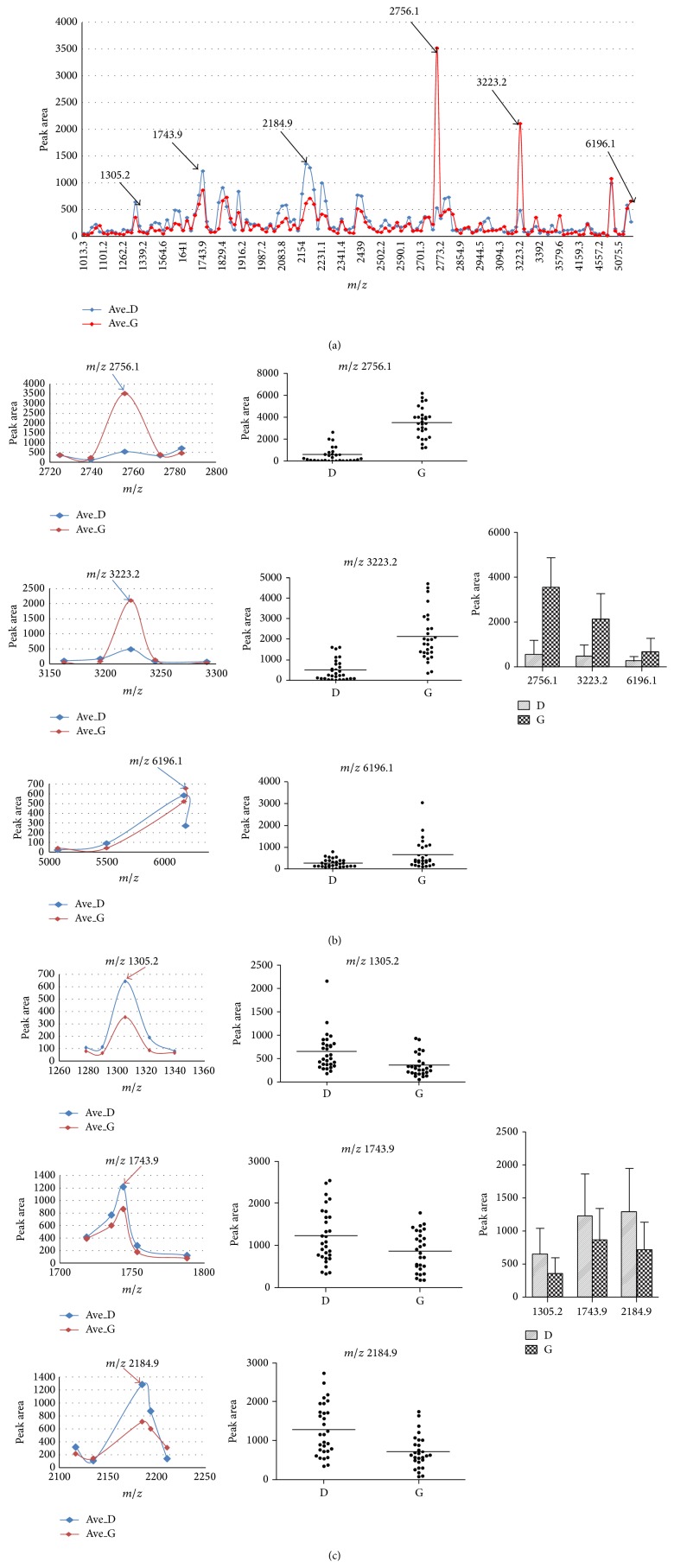
The feature of the 6 selected peaks in two groups. (a) The distribution of average peak area from two groups. (b) The average value of 3 elevated peaks in G group when compared with D group (left and right) and their distribution in all samples (middle). (c) The average value of 3 decreased peaks in G group when compared with D group (left and right) and their distribution in all samples (middle).

**Figure 3:**
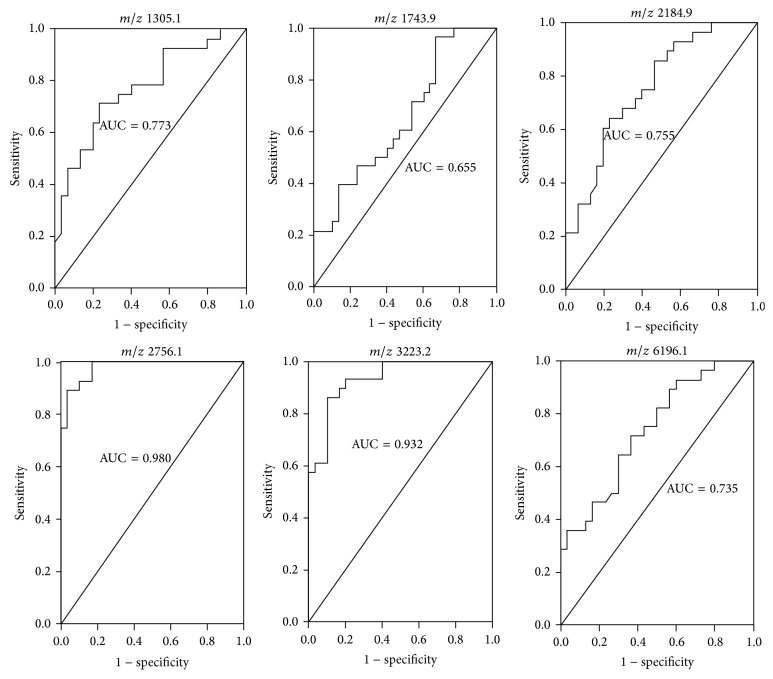
Receiver operator characteristics (ROC) curves generated with the* m/z* 1305.2, 1743.9, 2184.9, 2756.1, 3223.2, and 6196.1 used to distinguish type 2 diabetic patients coexisting with coronary artery disease from type 2 diabetic patients without coronary artery disease. The areas under the curve (AUC) were 0.773, 0.655, 0.755, 0.980, 0.932, and 0.735 for the above mentioned peaks, respectively.

Using ClinprotTools2.1, a total of 139 distinguishable peaks were detected within the 1,000 to 10,000* m/z* range, with 90 peaks having differential expression and statistical significance *P* < 0.05. To avoid bias, we picked six relative higher peaks for further analysis and the mass-to-charge ratio of the six peaks was 1305.2, 1743.9, 2184.9, 2756.1, 3223.2, and 6196.1 (Figure 2(a)). Compared to D group,* m/z* 2756.1, 3223.2, and 6196.1 were upregulated (Figure 2(b)) and* m/z* 1305.2, 1743.9, and 2184.9 were downregulated in G group (Figure 2(c)). The statistical characteristics of the six selected peaks are shown in Table 2. To evaluate the diagnostic efficacy of these peptides, the ROC analysis was performed to calculate the sensitivities, specificities, and accuracies at different cut-off points for differentiating CAD type 2 diabetic patients from control subjects. In the ROC curves,* m/z* 2756.1 and 3223.2 had excellent area under the curve (AUC) values (0.98 and 0.93) which indicate a highly accurate diagnostic test, and* m/z* 6196.1, 1305.2, and 2184.9 had limited clinical utility AUC of 0.73, 0.773, and 0.755, respectively, while* m/z* 1743.9 had an AUC of 0.655 that suggests low diagnostic accuracy (Figure 3).

### 3.3. Identification of the Potential Urinary Biomarkers for CAD Patients with Coexisting Type 2 Diabetes

With this bead-based proteomic technology, we found six potential biomarkers for CAD patients with coexisting type 2 diabetes. The peptide sequence of the six differential peaks was identified by a nanoliquid chromatography-tandem mass spectrometry, and the Sequest search reported the protein name. Following MS/MS, the sequence of* m/z* 1305.2 was parsed as A.DSGEGDFLAEGGGV.R and it is a fragment of isoform 1 of fibrinogen alpha chain precursor. Similarly,* m/z* 1743.9 was parsed as K.MADEAGSEADHEGTHST.K and* m/z* 2756.1 was S.SYSKQFTSSTSYNRGDSTFESKSY.K and both of them are fragment of fibrinogen alpha chain precursor. The* m/z* 2184.9 comes from interalpha-trypsin inhibitor heavy chain H4 and its amino acid sequence is S.RQLGLPGPPDVPDHAAYHPF.R. The* m/z* 3223.2 is a fragment of prothrombin precursor and its amino acid sequence is C.GLRPLFEKKSLEDKTERELLESYIDGR.I. Unfortunately, the* m/z* 6196.1 peak sequence was not identified. The detailed results are shown in Table 3.

## 4. Discussion

Urine as a promising source of biomarkers identification associated with disease has recently been discussed and reviewed, especially when combined with proteomics [11], including an internationally harmonized urine collection protocol. Urinary proteomics, which would yield information pertinent to the function of both renal and extrarenal organs, was not only used in the urologic and genital diseases [6, 12], but also in other system diseases, like endocrine system and digestive system [10, 13]. Driven by the advancements in technology, mass spectrometry- (MS-) based proteomic studies aiming at defining clinically relevant biomarkers have been increasing in number. However, not all MS-analytical platforms used for biomarker discovery are suitable for clinical diagnostic applications. Common approaches of MS-based proteomics for clinical diagnosis include 2DE-MS, SELDI-MS, liquid chromatography- (LC-) MS, and capillary electrophoresis- (CE-) MS [14]. In view of urine containing low molecular mass proteome that does not undergo any significant change even when urine was stored for up to 3 days at 4°C or 6 h at room temperature [8, 9], so study of low mass protein/peptide may provide a new field for biomarker discovery. On top of this, we focus our research on the bead-based MALDI-TOF mass spectrometry with its small sample sizes for analysis, high-throughput capability, exquisitely sensitive and high-resolution peptide detection, and monitoring of disease progression accurately [15–17]. We directly purified urinary protein/peptides using weak cationic-exchange magnetic beads without trypsinization and then develop a profile of urine proteome through MALDI-TOF-MS. By comparison with ClinProTools2.1 software, we determined several markers that differentiated type 2 diabetic patients with CAD from control samples. We selected several proteins/peptides biomarkers instead of single one because a single biomarker lacks specificity and perhaps does not exist.

Type 2 diabetes mellitus and CAD share several common characteristics in pathophysiology, including genetic and environmental factors, but type 2 diabetes with coexisting CAD takes on its own features. Insulin resistance and hyperglycemia are central features of type 2 diabetes mellitus, and they confer additional impairment for myocardial infection [18]. The underlying molecular mechanism of this kind of cardiac dysfunction is still largely unknown, but it is thought to involve causal alterations in gene and protein expression [19]. Proteomic technology now allows us to examine differential alterations in protein expression in the disease group and its controls.

In our bead-based urinary proteomic study, we found several urinary peptides patterns that were differently expressed in type 2 diabetic patients with CAD when compared with type 2 diabetic patients without CAD.

The downregulated peptides* m/z* 1305.1 and* m/z* 1743.9 and the upregulated peptide* m/z* 2756.1 were different fragments of fibrinogen alpha chain precursor. From our identified results we can see that different peptides can come from the same protein, but they play various roles in diagnosis of disease. Fibrinogen, synthesized by the liver, is a major plasma protein that consists of pairs of 3 different polypeptide chains *α*, *β*, and *γ*, joined by disulfide bonds to form a symmetric dimeric structure. It is directly involved in the clotting process as a clotting factor and releases two fibrinopeptides A and B from the NH_2_ terminus of the *α*, *β* chains cleavage by thrombin. There are some other fragments [20] released from fibrin that degraded by plasmin. So the changes of the level of plasma fibrinogen or fragments from fibrinogen could represent some life state that associates with hypercoagulability such as cardiovascular and cerebrovascular thrombotic diseases [21, 22] and renal failure [23]. The peptide* m/z* 1305.1 is fibrinopeptide A (sites 20–35) and* m/z* 1743.9 is just a fragment (sites 602–620) released from fibrinogen *α* chain. Alkjaerdsig and Fletcher had reported catabolism and excretion of fibrinopeptide A [24]. Plasma fibrinopeptide A had been a sensitive marker of in vivo fibrin formation and was significantly increased in type 2 diabetic patients with vascular complications [25]. In our study, the fragments* m/z* 1305.1 and 1743.9 of fibrinogen alpha chain in urine were significantly decreased and* m/z* 2756.1 was increased in type 2 diabetic patients with CAD. The former has longer duration of diabetes and thus is exposed longer in the hyperglycemia that could lead to protein metabolic disorder and dyslipidemia. The difference exhibited by the urinary proteomics may be able to explain the complex metabolism under the hyperglycemia.

Inter-trypsin inhibitor heavy chain H4 (ITIH4) from which the* m/z* 2184.9 peptide is derived is a plasma kallikrein-sensitive glycoprotein (120 kDa) [26] that is expressed mainly in liver and that acts as an acute-phase protein [27]. The urinary peptide we identified as a biomarker is a disease-associated fragment including cancer and inflammatory disease that had been discovered in the plasma [28]. There is increasing evidence that oxidative stress and inflammation play critical roles in the pathogenesis of type 2 diabetes mellitus and the development of its complications. Interestingly, several papers have suggested that ITIH4 may represent clinically surrogate markers for the detection and classification of different disease types [28, 29]. However, the exact biological function of ITIH4 in vivo remains unclear.

In total there were three elevated peptides in the complication group compared with the other group. Unfortunately, one of the three urinary peptides* m/z* 6196.1 was not identified. As mentioned above, the* m/z* 2756.1 peak sequence was identified as fibrinogen alpha chain. The other one peptide* m/z* 3223.2 was fragment of prothrombin precursor. In either diabetes mellitus patients or CAD patients, a hypercoagulable state is associated with the increase in thrombosis. Elevated level of coagulation factors is suggested to contribute to hypercoagulability and is considered one of the risk factors that play an important part in the development of stroke and myocardial infarction [30]. Its elevated expression in urine may be the result of cardiac injury by hyperglycemia and so it may be a risk signal for the diabetic patients accompanied with CAD. From the AUC of peptides* m/z* 2756.1 and* m/z* 3223.2, we can conclude that they had a higher diagnostic efficacy for these diabetic patients with CAD.

In conclusion, the urinary peptides hold important information that may have direct clinical utility for disease diagnosis and classification. We are very interested in these peaks and our next plan will go further to research for every urinary peptide. However, it is a long way to go to introduce this technology into clinical practice.

## Figures and Tables

**Table 1:** Demographics and clinical characteristics of two groups of type 2 diabetes mellitus.

	D	G	*P* value
	(*n* = 30)	(*n* = 28)	
Gender (M/F)	22/8	15/13	0.118
DM (year)	4.80 ± 4.90	7.81 ± 7.06	0.040
CAD (year)	/	5.25 ± 4.05	/
Mean age	53.1 ± 9.9	67.0 ± 9.9	0.000
FPG (mmol/L)	9.12 ± 1.82	8.89 ± 2.23	0.605
HbA1c%	7.66 ± 1.40	7.56 ± 1.01	0.716
HbA1c (mmol/mol)	60.2 ± 15.3	59.1 ± 11.1	0.716
Cholesterol (mmol/L)	5.46 ± 1.10	4.70 ± 1.06	0.004
HDL (mmol/L)	1.38 ± 0.56	1.32 ± 0.28	0.577
LDL (mmol/L)	3.41 ± 0.90	2.65 ± 0.92	0.001
TRIG (mmol/L)	1.51 ± 0.59	1.50 ± 1.05	0.960
A/Cr (mg/g)	11.86 ± 7.05	13.48 ± 6.69	0.371

Data is presented as mean ± SD unless otherwise indicated.

D: type 2 diabetes without coronary artery disease; G: type 2 diabetes coexisting with coronary artery disease; M/F: male/female; DM: the duration of type 2 diabetes; CAD: the history year of coronary artery disease; FPG: fasting plasma glucose; HDL: high density lipoprotein; LDL: low density lipoprotein; TRIG: triglyceride; A/Cr: albumin/creatine.

**Table 2:** The statistics characteristic of the six selected peaks.

*m*/*z*	*P* value	D	G
1305.2	3.66*E* − 04	639.7 ± 396.3	354.7 ± 231.3
1743.9	0.02	1219.3 ± 641.4	863.4 ± 474.1
2184.9	2.93*E* − 04	1281.1 ± 667.2	708.7 ± 428.1
2756.1	<1*e* − 6	531.8 ± 662.7	3516.8 ± 1363.3
3223.2	<1*e* − 6	485.4 ± 499.1	2104.8 ± 1171.6
6196.1	0.002	269.4 ± 190.1	656.7 ± 645.6

The peak area of every peak in two groups is presented as mean ± SD. *P* value was calculated by *t*-test (normally distributed continuous data) or Wilcoxon test (nonnormally distributed continuous data). *P* < 0.05 was accepted as statistically significant difference. D: type 2 diabetes mellitus without coronary artery disease; G: type 2 diabetes coexisting with coronary artery disease.

**Table 3:** Identified peptides sequence of the selected peaks.

*m*/*z*	Molecular weight	Amino sequences	Protein name
1305.2	1309.55	A.DSGEGDFLAEGGGV.R	Isoform 1 of fibrinogen alpha chain precursor
1743.9	1744.67	K.MADEAGSEADHEGTHST.K	Fibrinogen alpha chain precursor
2184.9	2181.92	S.RQLGLPGPPDVPDHAAYHPF.R	Interalpha-trypsin inhibitor heavy chain H4
2756.1	2756.22	S.SYSKQFTSSTSYNRGDSTFESKSY.K	Isoform 1 of fibrinogen alpha chain precursor
3223.2	3220.74	C.GLRPLFEKKSLEDKTERELLESYIDGR.I	Prothrombin precursor
6196.1	Identification failure		

## Abbreviations

HbA1C: Hemoglobin A1c
MALDI-TOF MS: Matrix-assisted laser desorption ionization time-of-flight mass spectrometry
MB-WCX: Weak cationic-exchange magnetic beads
*m/z*: Mass-to-charge ratio
CAD: Coronary artery disease
FGA: Fibrinogen alpha chain
ITIH4: Interalpha-trypsin inhibitor heavy chain H4
MS: Mass spectrometry
2DE-MS: Two-dimensional gel electrophoresis-mass spectrometry
SELDI-MS: Surface enhanced laser desorption/ionization-mass spectrometry
ROC: Receiver operating characteristic curve.

## References

[1] Spencer E. A., Pirie K. L., Stevens R. J. (2008). Diabetes and modifiable risk factors for cardiovascular disease: the prospective Million Women Study. *European Journal of Epidemiology*.

[2] Sarwar N., Gao P., Seshasai S. R. (2010). Diabetes mellitus, fasting blood glucose concentration, and risk of vascular disease: a collaborative meta-analysis of 102 prospective studies. *The Lancet*.

[3] Grodos D., Tonglet R. (1994). Scandinavian simvastatin study (4S). *The Lancet*.

[4] Kannel W. B., McGee D. L. (1979). Diabetes and cardiovascular disease. The Framingham study. *The Journal of the American Medical Association*.

[5] Adachi J., Kumar C., Zhang Y., Olsen J. V., Mann M. (2006). The human urinary proteome contains more than 1500 proteins, including a large proportion of membrane proteins. *Genome Biology*.

[6] Lei T., Zhao X., Jin S., Meng Q., Zhou H., Zhang M. (2013). Discovery of potential bladder cancer biomarkers by comparative urine proteomics and analysis. *Clinical Genitourinary Cancer*.

[7] Pisitkun T., Johnstone R., Knepper M. A. (2006). Discovery of urinary biomarkers. *Molecular and Cellular Proteomics*.

[8] Schaub S., Wilkins J., Weiler T., Sangster K., Rush D., Nickerson P. (2004). Urine protein profiling with surface-enhanced laser-desorption/ionization time-of-flight mass spectrometry. *Kidney International*.

[9] Theodorescu D., Wittke S., Ross M. M. (2006). Discovery and validation of new protein biomarkers for urothelial cancer: a prospective analysis. *The Lancet Oncology*.

[10] Chu L., Fu G., Meng Q., Zhou H., Zhang M. (2013). Identification of urinary biomarkers for type 2 diabetes using bead-based proteomic approach. *Diabetes Research and Clinical Practice*.

[11] Decramer S., de Peredo A. G., Breuil B. (2008). Urine in clinical proteomics. *Molecular and Cellular Proteomics*.

[12] Chen J., Chen L.-J., Xia Y.-L. (2013). Identification and verification of transthyretin as a potential biomarker for pancreatic ductal adenocarcinoma. *Journal of Cancer Research and Clinical Oncology*.

[13] Xiao D., Meng F. L., He L. H., Gu Y. X., Zhang J. Z. (2011). Analysis of the urinary peptidome associated with *Helicobacter pylori* infection. *World Journal of Gastroenterology*.

[14] Fliser D., Novak J., Thongboonkerd V. (2007). Advances in urinary proteome analysis and biomarker discovery. *Journal of the American Society of Nephrology*.

[15] Ransohoff D. F. (2004). Rules of evidence for cancer molecular-marker discovery and validation. *Nature Reviews Cancer*.

[16] Qiu F., Liu H.-Y., Zhang X.-J., Tian Y.-P. (2009). Optimization of magnetic beads for MALDI-TOF MS analysis. *Frontiers in Bioscience*.

[17] Qiu F., Liu H. Y., Dong Z. N., Feng Y. J., Zhang X. J., Tian Y. P. (2009). Searching for potential ovarian cancer biomarkers with matrix-assisted laser desorption/ionization time-of-flight mass spectrometry. *The American Journal of Biomedical Sciences*.

[18] Dutka D. P., Pitt M., Pagano D. (2006). Myocardial glucose transport and utilization in patients with type 2 diabetes mellitus, left ventricular dysfunction, and coronary artery disease. *Journal of the American College of Cardiology*.

[19] McGregor E., Dunn M. J. (2006). Proteomics of the heart: unraveling disease. *Circulation Research*.

[20] Bootle-Wilbraham C. A., Tazzyman S., Marshall J. M., Lewis C. E. (2000). Fibrinogen E-fragment inhibits the migration and tubule formation of human dermal microvascular endothelial cells in vitro. *Cancer Research*.

[21] Shlipak M. G., Ix J. H., Bibbins-Domingo K., Lin F., Whooley M. A. (2008). Biomarkers to predict recurrent cardiovascular disease: the Heart and Soul Study. *The American Journal of Medicine*.

[22] Zoccali C., Mallamaci F., Tripepi G. (2003). Fibrinogen, mortality and incident cardiovascular complications in end-stage renal failure. *Journal of Internal Medicine*.

[23] Muntner P., He J., Astor B. C., Folsom A. R., Coresh J. (2005). Traditional and nontraditional risk factors predict coronary heart disease in chronic kidney disease: results from the atherosclerosis risk in communities study. *Journal of the American Society of Nephrology*.

[24] Alkjaerdsig N., Fletcher A. P. (1982). Catabolism and excretion of fibrinopeptide-A. *Blood*.

[25] Librenti M. C., D'Angelo A., Micossi P., Garimberti B., Mannucci P. M., Pozza G. (1985). *β*-Thromboglobulin and fibrinopeptide a in diabetes mellitus as markers of vascular damage. *Acta Diabetologica Latina*.

[26]  Pu X. P., Iwamoto A., Nishimura H., Nagasawa S. (1994). Purification and characterization of a novel substrate for plasma kallikrein ( PK-120) in human plasma. *Biochimica et Biophysica Acta*.

[27] Piñeiro M., Alava M. A., González-Ramón N. (1999). ITIH4 serum concentration increases during acute-phase processes in human patients and is up-regulated by interleukin-6 in hepatocarcinoma HepG2 cells. *Biochemical and Biophysical Research Communications*.

[28] Song J., Patel M., Rosenzweig C. N. (2006). Quantification of fragments of human serum inter-*α*-trypsin inhibitor heavy chain 4 by a surface-enhanced laser desorption/ionization-based immunoassay. *Clinical Chemistry*.

[29] Villanueva J., Shaffer D. R., Philip J. (2006). Differential exoprotease activities confer tumor-specific serum peptidome patterns. *The Journal of Clinical Investigation*.

[30] Ceriello A., Pirisi M., Giacomello R. (1994). Fibrinogen plasma levels as a marker of thrombin activation: new insights on the role of fibrinogen as a cardiovascular risk factor. *Thrombosis and Haemostasis*.

